# Intensity modulated radiation therapy (IMRT) for sinonasal tumors: a single center long-term clinical analysis

**DOI:** 10.1186/s13014-016-0595-9

**Published:** 2016-02-04

**Authors:** Vasileios Askoxylakis, Pia Hegenbarth, Carmen Timke, Ladan Saleh-Ebrahimi, Juergen Debus, Falk Röder, Peter E. Huber

**Affiliations:** CCU Mol&Radiation Oncology, German Cancer Research Center (DKFZ), 280 INF, Heidelberg, 69120 Germany; Department of Radiation Oncology, University of Heidelberg, Heidelberg, Germany; Department of Radiation Oncology, Malteserkrankenhaus, Flensburg, Germany; Department of Radiation Oncology, University Hospital of Munich (LMU), Munich, Germany

**Keywords:** Sinonasal tumor, Intensity modulated radiation therapy

## Abstract

**Background:**

Radiotherapy has a central role in the treatment of sinonasal malignancies, either as postoperative or as primary therapy. To study the efficacy and safety of intensity modulated radiotherapy (IMRT) for sinonasal tumors a single center retrospective evaluation focusing on survival and therapy related toxicity was performed.

**Methods:**

One hundred twenty two patients with primary (*n* = 82) or recurrent (*n* = 40) malignant sinonasal tumors were treated with intensity modulated radiotherapy between 1999 and 2009 at the University Clinic of Heidelberg and the German Cancer Research Center and retrospectively analyzed. Most patients had adenoid cystic carcinomas (*n* = 47) or squamous cell carcinoma (*n* = 26). 99 patients received postoperative radiotherapy. The median total dose was 64 Gy in conventional fractionation (1.8–2 Gy). Overall survival (OS), progression free survival (PFS) and local recurrence free survival (LRFS) rates were calculated using the Kaplan-Meier method. The log-rank test and Fishers Exact test were applied for univariate analysis, Cox-regression was used for multivariate analysis.

**Results:**

Median follow up was 36 months. 1-, 3- and 5-year estimated overall survival rates were 90, 70 and 54 % respectively. Median progression free survival and local recurrence free survival was 45 and 63 months respectively. Progression free survival and local recurrence free survival at 1, 3 and 5 years were 76, 57 and 47, and 79, 60 and 51 % respectively. 19 patients (15.5 %) were diagnosed with distant metastases. Univariate analysis revealed significantly improved OS and LRFS for treatment of tumors after primary diagnosis, first series of irradiation and radiation dose ≥60 Gy. Multivariate analysis revealed only treatment in primary situation as an independent prognostic factor for OS and LRFS. Acute CTC grade III mucositis was seen in 5 patients (4.1 %) and CTC grade II dysgeusia in 19 patients (15.6 %). Dysgeusia, dysosmia and ocular toxicity were the most common late adverse events.

**Conclusions:**

Our data support the results of previous studies and indicate that intensity modulated radiation therapy (IMRT) represents an effective and safe treatment approach for patients with sinonasal carcinomas.

## Background

Sinonasal tumors are uncommon malignancies, representing about 0.2–1 % of all cancers and 3–5 % of all upper respiratory tract tumors. The disease is characterized by a high heterogeneity both in primary site and histology. The most common histologies include the keratinizing or non-keratinizing squamous cell carcinomas, followed by adenoid-cystic carcinomas and adenocarcinomas, whereas neuroectodermal and neuroendocrine tumors, as well as soft tissue tumors may also occur [1].

Primary treatment of the sinonasal tumors is a surgical resection. Besides open surgery, technical advances in the last decades have allowed an effective and safe endoscopic resection [2]. Whereas early stage disease can be effectively treated with surgery, malignant sinonasal tumors are often asymptomatic, resulting in diagnosis at advanced stages. Due to anatomic characteristics of the head and neck region and the close proximity of the sinonasal tract to the cranial cavity, tumors often infiltrate critical adjacent structures, limiting a complete tumor resection with organ preservation.

Besides surgery, radiotherapy is a treatment option of increasing relevance and significance. Radiotherapy can be applied either as primary treatment for inoperable tumors, or as postoperative therapy. The combination of radiotherapy with surgery is superior, compared to radiation alone [3]. Complete surgical resection with postoperative radiation therapy is considered the mainstay of sinonasal cancer treatment [4]. Early studies revealed that already adjuvant conventional radiotherapy to maximal surgical resection leads to 5-year overall survival of about 40 % [5]. However, conventional radiotherapy has been associated either with incomplete target coverage or severe toxicity. Due to the close proximity to critical structures, such as optic nerves, eyes and retina, radiation-induced blindness, retinopathy and neuropathy were common adverse effects after conventional radiotherapy [6].

These severe toxicities have been the most limiting factors for radiation treatment in the past, emphasizing the necessity for the evaluation of modern radiotherapy techniques that allow for a homogeneous dose distribution in the tumor, while sparing the healthy surrounding tissues at the same time. Intensity modulated radiation therapy (IMRT) allows steep dose gradients close to the target and represents an effective method to optimize treatment planning of head and neck cancers and to deliver higher doses to the target, while minimizing the doses to the organs-at risk [7–9].

Aim of the current study was to present the results of a retrospective analysis of 122 consecutive patients with sinonasal malignancies, who received intensity modulated radiotherapy in our institution. Overall survival, progression free- and local recurrence free survival, and radiation-induced toxicity were analyzed to generate information that will facilitate prospective trials focusing on the role of modern radiation therapy approaches in the treatment of sinonasal cancer.

## Methods

### Patients

122 adult patients with sinonasal tumors treated between 1999 and 2009 with intensity modulated radiotherapy in the Department for Radiation Oncology at the University Clinic of Heidelberg and the German Cancer Research Center were included in our analysis. The patients’ medical records and follow-up data were retrospectively evaluated. Analysis included gender, age, histology, staging, resection margin, tumor status at IMRT (primary vs recurrent situation at presentation), radiation series (first series vs re-irradiation), local recurrence free survival (LRFS), progression free survival (PFS) and overall survival (OS). Radiation-induced toxicity analysis included acute (<90 days) and late (>90 days) adverse effects. The patients’ characteristics are presented in Table 1.

**Table 1:** Patients characteristics

Clinical characteristics	Number (*n*)	Percent
Total number	122	100
Age at diagnosis (years)		
Median	56	
Range	21–79	
Gender		
Male	74	61
Female	48	39
Histology		
Adenoid cystic	47	38.6
Squamous cell	26	21.3
Adenocarcinoma	17	13.9
Soft tissue sarcoma	7	5.7
Melanoma	6	4.9
Undifferentiated	4	3.3
Other	15	12.3
Tumor stage		
T1	12	9.8
T2	7	5.7
T3	16	13.2
T4	87	71.3
Primary tumor site		
Nasal sinuses	86	70.5
Nasal cavity	36	29.5
Tumor state at IMRT		
Primary diagnosis	82	67.2
Recurrence	40	32.8
Resection status		
R0	11	9.0
R1	24	19.7
R2	33	27.1
Rx	31	25.4
No resection	23	18.8
Concurrent Systemic therapy		
None	108	88.5
Chemotherapy	11	9.0
Cetuximab	3	2.5
RT dose (Gy)		
≥ 60 Gy	87	71.3
< 60 Gy	35	28.7

### Surgery/chemotherapy

99 patients (81.1 %) underwent surgical resection before radiotherapy. 23 patients (18.9 %) received primary radiotherapy. 27 patients received either neoadjuvant or simultaneous mainly platin-based chemotherapy. Three patients received cetuximab simultaneously to IMRT.

### Radiotherapy

All patients received intensity modulated radiotherapy (IMRT). IMRT was applied using the step and shoot approach as previously described [9]. Patient immobilization was performed using a Scotch Cast (3 M, St Paul, Minneapolis, MN) head mask, which allows a setup accuracy of 1–2 mm. For treatment planning contrast-enhanced CT- and MRI-images were carried out in the immobilization system. Stereotactic image fusion was performed and the target volumes and organs-at-risk were defined on each slice. The Gross Tumor Volume (GTV) included the macroscopic tumor visible on CT- and MRI-scans. A safety margin of 3–5 mm was added to create a Boost CTV and another 3 mm were usually added to create a Boost PTV. A second Clinical Target Volume included the GTV (if present) and all involved or surgically affected paranasal sinus and the nasal cavity. Usually a safety margin of 3 mm was added to receive the second PTV. In patients without gross disease, the only CTV and PTV were created as described as the second PTV in patients with gross disease. Margins could be reduced on the discretion of the treating radiation oncologist in case of directly adjacent organs at risk with low radiation tolerance.

Treatment planning was carried out as inverse planning using the Konrad software, developed at the German Cancer Research Center, Heidelberg, as previously described [10]. Treatment was delivered by linear accelerator with 6 or 15 MeV photons using an integrated multileaf collimator (MLC).

The total dose was prescribed to the median of the target volume taking into account the tolerance doses of the adjacent organs-at-risk after treatment planning and analysis of the dose volume histograms (DVH). Dose constraints were set at 54 Gy for the optic nerves, brainstem and chiasm, 26 Gy (mean dose) for the parotid glands and 45 Gy for the spinal cord. A summary of the median and maximal doses at OAR is presented in Table 2.

**Table 2:** Summary of the DVH data

Characteristics	Mean	SE	Median	Range
GTV				
D_med_ (Gy)	61.33	0.77	64	(34.92–73.01)
D_max_ (Gy)	71.85	0.93	73.05	(39.13–95.7)
Brain Stem				
D_med_ (Gy)	22.67	0.79	20.86	(0.86–40.43)
D_max_ (Gy)	44.41	1.25	47.39	(2.38–69.55)
Chiasm				
D_med_ (Gy)	24.79	0.84	24.29	(2.43–42.37)
D_max_ (Gy)	40.45	1.14	42.17	(3.38–66.38)
Right optic nerve				
D_med_ (Gy)	37.32	1.03	38.94	(0.58–62.5)
D_max_ (Gy)	49.23	1.11	51.56	(0.65–75.24)
Left optic nerve				
D_med_ (Gy)	35.90	1.01	36.17	(2.97–60.85)
D_max_ (Gy)	47.52	1.05	49.24	(3.37–74.38)
Right eye				
D_med_ (Gy)	22.10	0.88	19.70	(0.83–42.97)
D_max_ (Gy)	43.58	1.25	43.63	(4.07–69.37)
Left eye				
D_med_ (Gy)	21.96	0.95	20.36	(1.63–48.97)
D_max_ (Gy)	41.56	1.32	42.57	(4.46–70.22)
Spinal cord				
D_med_ (Gy)	10.75	0.77	8.13	(0.24–29.05)
D_max_ (Gy)	29.75	1.26	33.26	(1.72–57.24)

The median dose prescribed to the boost PTV in case of gross disease was 64 Gy. In these patients the median dose prescribed to the second PTV was 54 Gy. In patients without gross disease, the median dose prescribed to the only PTV was 59.4 Gy. 49 patients received an integrated boost irradiation. In 15 cases IMRT was performed as re-irradiation. IMRT was performed at a median fractionation of 2 Gy (1.8–2.2 Gy), 5 days per week.

### Follow up

Patient follow up was performed at 6–8 weeks post radiation treatment and then every 3 months for the first 2 years, every 6 months for the following 3 years and annually thereafter. Follow-up included medical history and physical examination, followed by MRI- or CT-Scan. In addition, follow-up included interdisciplinary examinations by otorhinolaryngologists and opthalmologists at a regular basis.

### Data analysis

Overall survival (OS) was defined from the day of IMRT begin to the time of death from any cause or last follow up. Progression free survival (PFS) was defined from the day of IMRT begin to the day of local or distant disease recurrence, diagnosed by imaging examinations. Local recurrence free survival (LRFS) was defined from the day of treatment begin to the day of local relapse. Acute (<90 days after IMRT initiation) and late (>90 days after IMRT initiation) radiation-induced toxicity was scored according to the Common Toxicity Criteria (CTC) version 3.0 of the U.S. National Institutes of Health.

### Statistics

Statistical analysis was performed using Prism (GraphPad Software Inc., La Jolla, CA) or the Statistical Software STATA 13.1. Survival rates were calculated using the Kaplan-Meier method. Univariate analysis was performed using the log-rank test and Fishers Exact test. For multivariate analysis Cox-regression was applied. A *p* value <0.05 was considered statistically significant.

## Results

### Patient related parameters

Median patient age at first diagnosis and initiation of radiotherapy was 56 years (range, 21–79 years) and 58 years (range, 23–81) respectively. Median follow-up was 36 months (range, 1–124 months). Tumor stage at first diagnosis was T1 in 12 cases (9.8 %), T2 in 7 (5.7 %), T3 in 16 (13.2 %) and T4 in 87 cases (71.3 %). Histology analysis revealed adenoid cystic carcinoma (*n* = 47, 38.5 %), squamous cell carcinoma (*n* = 26, 21.3 %) and adenocarcinoma (*n* = 17, 13.9 %) as main histologies. A complete R0 resection was achieved in 11 cases (9 %). R1 resection was confirmed in 24 cases (19.7 %), whereas 33 patients (27 %) received a debulking surgery and 23 (19 %) patients had no surgery at all. In 31 patients (25 %) no definitive resection status could be established because of unknown marginal status, mainly due to fragmented resection. 82 patients (67.2 %) received IMRT for the treatment of tumor at primary diagnosis, whereas 40 patients (37.8 %) received radiotherapy for the treatment or recurrent disease. Patients’ characteristics are presented in Table 1.

### Dosis distribution

The median and maximal dose in organs at risk was evaluated and the means were calculated. 12 patients (9.8 %) received a maximal dose >45 Gy in the spinal cord. 51 and 61 patients (41.8 and 50 %) had a maximal dose >50 Gy in the left and right optic nerve respectively. 28 patients received a maximal dose >50 Gy to the optical chiasm. In 63 cases (51.6 %) a maximal dose >45 Gy was delivered to the brainstem. The dose distribution in organs at risk is presented in Table 2.

### Survival results

**Fig. 1:**
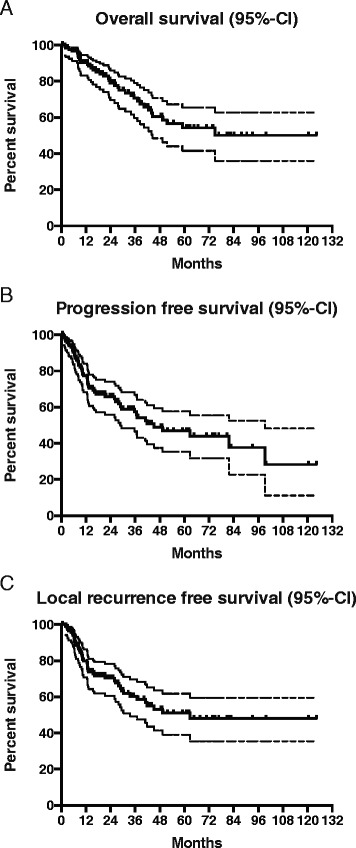
Kaplan-Meier estimates for survival in all patients. **a** Overall survival, **b** progression free survival, **c** local recurrence free survival

One- and 3-year survival rates were 90 and 70 % respectively. Estimated 5-year survival rate was 54 %. Median progression free survival (PFS) was 45 months and median local-recurrence free survival (LRFS) was 63 months. PFS and LRFS rates at 1 and 3 years were 76, 57 and 79 %, 60 % respectively. Estimated 5-year PFS and LRFS were 47 and 51 % respectively. The Kaplan-Meier estimates for OS, PFS and LRFS are presented in Fig. 1.

Investigation of differences in OS between various groups was performed using the log-rank test and the Fishers Exact test. Survival outcome was separately investigated for patients with squamous cell carcinoma (SCC) and adenoid cystic carcinoma (ACC). 1-, 3- and 5-years OS rates were 88, 68 and 56 % for SCC and 91, 77 and 60 % for ACC (*p* = 0.69). Comparison of SCC or ACC with any other histology did not reveal any statistically significant difference (*p* = 0.89 and *p* = 0.76 respectively).

**Fig. 2:**
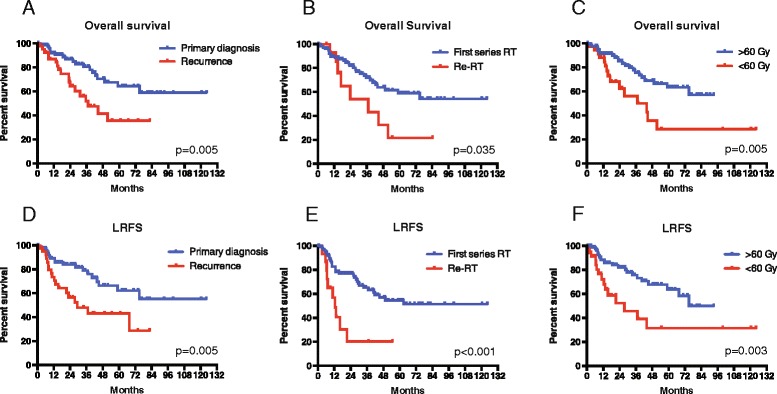
Kaplan Meier estimates of overall survival (OS) and local recurrence free survival (LRFS) in subgroups that were significantly different in univariate analysis. **a** OS for primary diagnosed versus recurrent tumors, **b** OS for patients who received first series of irradiation versus re-irradiation, **c** OS for ≥60 Gy versus <60 Gy, **d** LRFS for primary diagnosed versus recurrent tumors, **e** LRFS for first series of irradiation versus re-irradiation, **f** LRFS for ≥60 Gy versus <60 Gy

For patients who received IMRT for the treatment of tumors at primary diagnosis 1-,3- and 5-year overall survival rates were 92, 81 and 64 % respectively. The same rates for patients who received IMRT after disease relapse were 87, 52 and 36 % respectively. Log rank analysis revealed that this difference was statistical significant (*p* = 0.005, Fig. 2a). Furthermore, analysis was performed for patients who received a first series of irradiation versus re-irradiation. Log rank analysis revealed a significant improvement in overall survival for patients receiving a first series of irradiation versus re-irradiation (*p* = 0.035, Fig. 2b).

In regard to surgery, analysis focused on the resection status. For patients with R0 resection, the estimated 5- year OS rate was 91 %. 1-, 3- and 5-year OS rates were 91, 63 and 44 % for R1 and 91, 65 and 46 % for R2 resection status. Statistical analysis revealed no difference between R1 and R2 resection status (*p* = 0.9) but a trend to a statistically significant improvement of OS for patients after R0 surgery.

To evaluate the impact of radiation therapy dose to treatment outcome, overall survival was analyzed for the group of patients who received a radiation dose ≥60 Gy versus a dose <60 Gy. 1-, 3- and 5-years overall survival was 91, 76 and 63 % for the ≥60 Gy group and 88, 56 and 29 % for the <60 Gy group respectively. Log rank analysis revealed that this difference was statistically significant (*p* = 0.005, Fig. 2c).

Similar analysis was performed for local recurrence free survival. Univariate analysis revealed a statistically significant LRFS benefit for patients who received IMRT for the primary tumor (*p* = 0.005, Fig. 2d), patients who received first series of irradiation (*p* < 0.001, Fig. 2e) and for patients who received a radiation dose ≥60 Gy (*p* = 0.003, Fig. 2f). Furthermore, LRFS was improved for patients with ACC histology, compared to non-ACC histology (*p* = 0.026).

In addition, we performed multivariate analysis for OS and LRFS using Cox-regression. Multivariate analysis revealed only treatment in primary situation as an independent prognostic factor for both OS and LRFS (*p* = 0.004 and *p* = 0.008 respectively) (Tables 3 and 4).

**Table 3:** Multivariate analysis for overall survival

	Haz. Ratio	Std. Err	z	*P* > |z|	[95 % Conf. Interval]
Gender (male)	0.894	0.447	−0.22	0.823	[0.336–2.380]
Age (>65)	1.476	0.770	0.75	0.455	[0.532–4.100]
Histology	1.562	0.917	0.76	0.447	[0.495–4.933]
R-Status (R1/R2)	5.595	6.485	1.49	0.137	[0.577–54.249]
Primary diagnosis	0.236	0.119	−2.87	0.004	[0.088–0.633]
Dose (<60 Gy)	0.977	0.657	−0.04	0.972	[0.261–3.651]
RT series (first)	0.853	1.060	−0.13	0.898	[0.074–9.750]

**Table 4:** Multivariate analysis for local recurrence free survival

	Haz. Ratio	Std. Err.	z	*P* > |z|	[95 % Conf. Interval]
Gender (male)	0.944	0.433	−0.12	0.901	[0.384–2.321]
Age (>65)	1.300	0.665	0.51	0.608	[0.476–3.547]
Histology	1.351	0.662	0.61	0.540	[0.516–3.533]
R-Status (R1/R2)	4.365	4.742	1.36	0.175	[0.519–36.705]
Primary diagnosis	0.264	0.133	−2.64	0.008	[0.098–0.710]
Dose (<60 Gy)	0.963	0.536	−0.07	0.947	[0.323–2.869]

### Distant metastasis

Among 122 patients included in our analysis, 19 patients (15.5 %) developed distant metastases after IMRT. The majority of the cases with distant failure were diagnosed with lung metastases (9 patients, 7.3 %). The median time to the onset of distant metastases in patients with distant failure was 18.5 months (range, 1–99 months). Among the patients who developed distant metastases 12 patients had ACC and 7 non-ACC histology, which was statistically different (*p* = 0.02).

### Acute toxicity

Grade I/II acute dermatitis and mucositis were observed in 92 (74.4 %) and 42 (34.1 %) of the patients respectively. 1 patient had acute dermatitis Grade III and 5 patients had a Grade III mucositis. Xerostomia was observed in 29 patients (23.6 %). Among them only 2 patients had a Grade III xerostomia. The rates of dysphagia and dysgeusia were 21.3 and 31.2 % respectively. 2 patients had a Grade III dysphagia and received a percutaneous endoscopic gastrostomy. 60 patients were observed with ocular toxicity. The incidence of dry eye, conjunctivitis and tearing was 10.6, 18.7 and 15.4 % respectively. 5 patients had blurred vision and photophobia. An overview of acute toxicity post IMRT is presented in Table 5.

**Table 5:** Adverse events (non ocular)

Adverse Event	Grade I	Grade II	Grade III	Grade IV
Acute toxicity				
Mucositis	24 (19.7 %)	18 (14.8 %)	5 (4.1 %)	0
Dermatitis	77 (63.1 %)	15 (12.3)	1 (0.8 %)	0
Xerostomia	22 (18.0 %)	5 (4.1 %)	2 (1.6 %)	0
Dysphagia	15 (12.3 %)	9 (7.4 %)	2 (1.6 %)	0
Dysgeusia	19 (15.6 %)	19 (15.6 %)	-	-
Dysosmia	9 (7.4 %)	6 (4.9 %)	-	-

### Late toxicity

Late toxicity (>90 days post IMRT treatment) was assessed. Common late toxicity included dysgeusia or dysosmia (42 patients, 34.4 %). In 8 cases (6.6 %) Grade II dysosmia was diagnosed, whereas 11 patients (9 %) developed Grade II dysgeusia. Ocular late toxicity was observed in 42 patients (34 %). 22 patients (18 %) had chronic tearing, 3 patients (2.4 %) light sensitivity and 8 patients (6.6 %) vision reduction. 2 (1.6 %) patients developed a cataract. Xerostomia was observed in 16 cases (13.1 %).

## Discussion

The optimal local treatment for malignant sinonasal tumors is subject of intensive clinical investigation, mainly due to the fact that these tumors are often diagnosed at advanced stages and the chances of a complete resection are limited. Aim of the present study is to evaluate the impact of intensity modulated radiotherapy (IMRT) in patients with sinonasal malignancies. We performed a retrospective analysis of 122 cases that received IMRT, either in the postoperative setting, or as primary treatment. The 5-year estimate of overall survival was 54 %. The local recurrence rate estimate at 5 years was 49 %. Univariate analysis revealed a statistically improved survival for patients who were treated in primary situation, patients who received first series of irradiation and patients that received a radiation dose ≥60 Gy, as well as a trend for improved survival for patients who underwent complete tumor resection (R0) prior to radiotherapy. Univariate analysis further revealed an improved LRFS for patients with ACC histology. Multivariate analysis revealed only treatment in primary situation as an independent prognostic factor for OS and LRFS (Tables 3 and 4). Our study showed that 15.5 % of the patients developed distant metastases with the majority diagnosed with pulmonary metastases (7.3 %). Analysis of acute toxicity revealed mucositis to be the most common Grade III adverse effect.

5-year OS rates for patients with sinonasal tumors treated with IMRT varies in the literature between 16 and 52 %. Our data seem to be superior compared to early studies on IMRT for sinonasal tumors [11–13] and similar compared to more recent data [14–16]. This can be explained by improvements in both radiotherapy and surgery in the last years. However, the good outcome in our analysis might be also affected by the high ratio of patients with adenoid cystic carcinoma (47, 38.6 %). ACC is characterized by lower progression/growth rate and better outcome compared to squamous cell carcinoma or adenocarcinoma. The role of histological subtype on the outcome of radiotherapy has been extensively described in other studies. Hoppe et al. showed a decreased outcome of radiotherapy for SCC in a retrospective analysis of 85 patients who received postoperative radiotherapy [17]. Similar results were found in other studies [18]. However, in these studies many patients were treated with conventional or 3D-conformal radiotherapy and with lower median doses. In our study, no difference in overall survival was found between SCC and ACC, or ACC and any other histology. Considering that studies have described other histologic subtypes, such as undifferentiated tumors or melanoma to be associated with poor prognosis compared to ACC or adenocarcinoma [19, 20], we compared the outcome of patients with poor prognosis-associated histology (SCC or melanoma or undifferentiated carcinoma) with the outcome of patients with ACC or adenocarcinoma; our comparison revealed no difference between these groups in overall survival (*p* = 0.92).

In regard to the role of the resection status on the outcome of postoperative IMRT our analysis revealed no difference between R1-status and tumor debulking (R2-status). In patients classified as Rx the planning MRI did not reveal clear macroscopic residue. The Rx pathology might be associated with the challenge of determining the resection margins of sinonasal tumors, since in many cases these tumors cannot be resected en bloc, but rather in multiple fragments. The estimated 5-year overall survival was better after complete tumor resection. The result was not statistically significant, but this might be attributed to the low number of patients in the R0 group (11 cases).

Our univariate analysis demonstrated a strong effect of radiation dose and radiation series (first series of irradiation vs re-irradiation) on treatment outcome. In particular, survival was significantly improved for patients who received a first series of irradiation or a dose of ≥60 Gy. Similar results have been described in other studies [21]. In a study by Airoldi et al. [22], dose escalation had no significant effects on therapy outcome, however the cutoff in this study was set at 55.8 Gy. Despite the result of the univariate analysis, the radiation dose and the series or irradiation were not found to be an independent prognostic factor for survival in a multivariate analysis.

The only factor that was found to be of high significance in our multivariate Cox regression analysis was the state of the tumor at IMRT treatment (primary diagnosis vs recurrent disease). Tumors treated with IMRT after primary diagnosis had a significantly improved outcome. The poor outcome of recurrent tumors can be explained by various factors: i) 15 patients had relapsed after previous radiotherapy, allowing the hypothesis that there was a selection of radiation resistant tumors in this group; ii) in these 15 cases of relapsed tumors IMRT was performed as re-irradiation, which limits the dosis due to organ-at-risk tolerance, leading to lower doses of relapsed tumors compared to primarily diagnosed malignancies.

In regard to local control, the 5-year local recurrence free survival was 51 %. Univariate analyses revealed statistically improved local control for tumors receiveing IMRT after primary diagnosis (*p* = 0.005), tumors receiving first series of irradiation (*p* < 0.001) and dose ≥60 Gy (*p* = 0.003), whereas the multivariate analysis revealed only tumor status at treatment (newly diagnosed vs recurrent) to be an independent prognostic factor. Similar local control rates are described in the literature. In a study with 71 patients with sinonasal tumors, who received either a combinatorial therapy consisting of surgery and radiotherapy or a monotherapy, the local control rate at 5 years was about 59 %. A review and meta-analysis revealed local control rates of about 56 % [18]. Previous data revealed increased local recurrence rates for SCC, compared to ACC [17]. This might be attributed to a slower growth of ACC, leading to a later diagnosis of local recurrence. Despite the high incidence of ACC in our population, a difference to SCC could not be detected. However, local recurrence free survival was improved for ACC compared to any other histology. The discrepancy between LRFS and OS for ACC versus any other histology may be explained by the higher incidence of distant metastases in the ACC group in our study (*p* = 0.02). This has likely negatively affected the overall survival outcome in the ACC group.

Although local tumor relapse is the major problem in sinonasal malignancies, distant metastases can also occur. In our study, 19 patients (15.5 %) developed distant metastases post IMRT treatment. Distant metastases rates in patients with sinonasal tumors vary in the literature between 10 and 30 % [6, 18, 23]. However, it must be noted that the distant metastases rates might be underestimated due to the fact that patients did not have re-staging for distant metastases and in many cases the local relapse was the driver of the prognosis, leading to patient death before development of metastases-specific symptoms that would facilitate their diagnosis. The fact that local recurrence drives the prognosis of sinonasal tumors emphasizes the necessity for the optimization of local treatments and supports the rationale for combining maximal surgery with postoperative radiotherapy.

A major drawback in the use of radiotherapy for malignant sinonasal tumors is the treatment-related toxicity. Due to the close proximity of the tumors to critical organs at risk, such as optic nervs, optic chiasm, brainstem and eyes, conventional radiotherapy techniques are limited in the dose application and associated with severe adverse effects. Retinopathy and optic neuropathy occurred in up to 40–50 % of patients treated with conventional radiotherapy in the past [16]. IMRT provides the advantages of sparing the critical structures at risk while allowing greater conformity to the target. At a median follow-up of 36 months, we observed acute vision impairment and photophobia in 5 cases. Late vision impairment was diagnosed in 8 patients (6.5 %), whereas total late ocular toxicity was observed in 34 % of the patients. A relevant question that is raised is whether late ocular toxicity correlates with the relative high dose maximum in the eyes, the optic nerves or the optic chiasm. These high maximum doses are due to inhomogeneous dose distributions in IMRT and were applied at very small volumes. We investigated a possible correlation between maximum dose for each organ-at-risk and relevant late toxicity by comparing the maximum and mean doses in eyes, optic nerves and optic chiasm in the group of patients that did not show any ocular late toxicity and in the group of patients who did. Our analysis did not reveal any differences for the optic nerves and the optic chiasm. However, the maximum doses for the eyes were higher in the group of patients who presented with late ocular toxicities (*p* = 0.04 and *p* = 0.08 for the right and left eye respectively). This is explained by the fact that high maximum doses were the result of closer proximity of the tumor to the eyes and correlated with higher median doses in them. Acute Grade III mucositis and xerostomia were obsereved in 5 and 2 cases respectively. These results are comparable to other reports on IMRT for sinonasal tumors. In a recent anaylsis, Duprez et al. observed Grade III tearing in 10 cases and Grade III visual impairment in 1 case of 86 patients available for late toxicity evaluation (>6 months post IMRT) [16]. Other studies did not observe ≥ Grade III visual impairment [15, 24]. However, it should be noted that late radiation-induced ocular toxicity can develop even years post radiotherapy and, therefore, longer follow-up periods are necessary for safe conclusions.

Despite the important clinical information provided by the results of our study, there are some critical limitations that need to be considered. Our work is a retrospective analysis, and therefore selection bias might be facilitated. In addition, despite the fact that our study is one of the largest investigations on the role of IMRT for sinonasal tumors, the included number of patients is still low and might not allow safe statistical conclusions, emphasizing the necessity for cautious interpretations. Further prospective clinical trials are necessary in order to clearly define the impact of intensity modulated radiotherapy within multimodal therapeutic strategies, identify patients that will mostly benefit from IMRT and optimize local treatment of malignant sinonasal tumors.

## Conclusions

In conclusion, the presented data indicate that intensity modulated radiotherapy represents an effective and safe treatment approach for patients with malignant sinonasal tumors. IMRT allows the delivery of high radiation doses to the tumor, resulting in high local and distant control rates. At the same time IMRT facilitates the reduction of radiation-induced toxicity to critical organs at risk. Despite the relative large number of consecutive patients in the study, certain limitations need to be considered including the retrospective character of our work. Therefore, further analyses within prospective clinical trials could more clearly define the impact of IMRT and optimize treatment of sinonasal malignancies.

### Ethics approval

The study is in compliance with the Declaration of Helsinki (Sixth Revision, 2008). The study was approved by the Independent Ethics Committee of the Medical Faculty of the University of Heidelberg, Heidelberg, Germany (Ref. Nr.: S-489/2010).
